# Repeatability and Agreement of Central Corneal Thickness and Keratometry Measurements between Four Different Devices

**DOI:** 10.1155/2017/6181405

**Published:** 2017-03-05

**Authors:** Laszlo Kiraly, Jana Stange, Kathleen S. Kunert, Saadettin Sel

**Affiliations:** ^1^Augen- und Laserzentrum Leipzig, Lampestraße 1, 04107 Leipzig, Germany; ^2^Ernst-Abbe-Hochschule Jena, Carl-Zeiß-Promenade 2, 07745 Jena, Germany; ^3^Department of Ophthalmology, Heidelberg University, Im Neuenheimer Feld 400, 69120 Heidelberg, Germany

## Abstract

*Background.* To estimate repeatability and comparability of central corneal thickness (CCT) and keratometry measurements obtained by four different devices in healthy eyes. *Methods.* Fifty-five healthy eyes from 55 volunteers were enrolled in this study. CCT (IOLMaster 700, Pentacam HR, and Cirrus HD-OCT) and keratometry readings (IOLMaster 700, Pentacam HR, and iDesign) were measured. For statistical analysis, the corneal spherocylinder was converted into power vectors (J0, J45). Repeatability was assessed by intraclass correlation coefficient (ICC). Agreement of measurements between the devices was evaluated by the Bland-Altman method. *Results.* The analysis of repeatability of CCT data of IOLMaster 700, Pentacam HR, and Cirrus HD-OCT showed high ICCs (range 0.995 to 0.999). The comparison of CCT measurements revealed statistically significant differences between Pentacam HR versus IOLMaster 700 (*p* < 0.0001) and Pentacam HR versus Cirrus HD-OCT (*p* < 0.0001), respectively. There was no difference in CCT measurements between IOLMaster 700 and Cirrus HD-OCT (*p* = 0.519). The repeatability of keratometry readings (J0 and J45) of IOLMaster 700, Pentacam HR, and iDesign was also high with ICCs ranging from 0.974 to 0.999. The Pentacam HR revealed significantly higher J0 in comparison to IOLMaster 700 (*p* = 0.009) and iDesign (*p* = 0.041); however, no significant difference was between IOLMaster 700 and iDesign (*p* = 0.426). Comparison of J45 showed no significant difference between IOLMaster 700, Pentacam HR, and iDesign. These results were in accordance with Bland-Altman plots. *Conclusion.* In clinical practice, the devices analyzed should not be used interchangeably due to low agreement regarding CCT as well as keratometry readings.

## 1. Introduction

The precise knowledge of the corneal shape, refractive power, and corneal thickness is of great importance for the preparation and results of refractive and cataract surgery. For example, a minimum corneal thickness of 480 *μ*m is necessary to minimize the risk of ectasia after laser in situ keratomileusis (LASIK) [1]. Corneal curvature is required for all intraocular lens (IOL) formulas and thus influences the power of the selected IOL [2]. There are different types of technology for measuring corneal curvature that can be divided into Placido disc-based or slit-scanning-based method and Scheimpflug imaging [3, 4]. When it comes to determining corneal thickness, ultrasound pachymetry is regarded as the gold standard, because of its high degree of reproducibility [5]. However, this method leads to patient discomfort because corneal-probe contact is required, and in addition, the risk of infections increases [6]. Therefore, noncontact methods are preferred. Noncontact technology includes optical coherence tomography, low-coherence reflectometry, partial-coherence interferometry, and slit-scanning pachymetry. This wide range of options and the variety of measurement tools require quality comparison of these instruments.

The IOLMaster 700 (Carl Zeiss Meditec AG, Jena), which lately became commercially available, is a swept-source optical biometer to determine keratometry, central corneal thickness (CCT), anterior chamber depth, anterior aqueous depth, lens thickness, horizontal white-to-white corneal diameter, pupil size, and axial length within a single scan [7]. It is widely used for calculating biometric data before cataract surgery.

The Pentacam HR (OCULUS, Wetzlar, Germany) generates images of the anterior eye segment by the Scheimpflug principle. It produces a topographic map of the anterior and posterior corneal surfaces and anterior chamber and keratometry and pachymetry data.

The iDesign (iDesign System, Abbott Medical Optics Inc., USA) is a high-density wavefront aberrometer that is equipped with a Shack-Hartmann sensor and a corneal topographer. The system is able to measure wavefront aberrations, corneal topography, and keratometry.

The Cirrus HD-OCT (Carl Zeiss Meditec AG, Jena, Germany) is a spectral domain optical coherence tomography system that is capable of analyzing the anterior eye segment, retina, and optic nerve.

To our knowledge, there are no published data that evaluated the comparability of a swept-source OCT (IOLMaster 700) with a Scheimpflug imaging camera (Pentacam HR), a Hartmann-based topographer (iDesign), and a Fourier-domain OCT (Cirrus HD-OCT). In this prospective study, we analyze the repeatability and comparability of central corneal thickness (CCT) measurements using the new IOLMaster 700, Pentacam HR, and Cirrus HD-OCT and evaluate the repeatability and agreement of keratometry readings using the IOLMaster 700, Pentacam HR, and iDesign.

## 2. Methods

Fifty-five healthy volunteers of legal age (18 years and older) were recruited from the Eye and Laser Center, Leipzig, Germany, for this study. Informed consent was obtained from all subjects after explaining the nature of the study. The study included only normal eyes without any ophthalmological abnormalities, history of ocular pathology, or ocular surgery. All subjects were asked to remove their contact lenses 14 days prior to the study. To exclude any corneal pathology, a standardized clinical slit-lamp examination was performed on all participants. Using an automated keratorefractometer (Nidek AR-1; NIDEK CO., Japan), all participants were subjected to spherical refractive error. The study was performed according to the Declaration of Helsinki and Good Clinical Practices and was approved by the ethics committee at the Saxony Medical Council (EK-BR-68/15-1).

After patients signed informed consent, all measurements were taken in a single session. Patients were asked to place their chin on the chin rest and forehead against the forehead support and to look at the fixation point of the respective device as specified by the manufacturer. The measurements were performed according to the optical orientation of the device across the screen (e.g., centered on the pupil center), triggered manually via the control unit, except for the Pentacam HR. The Pentacam HR automatically started measuring as soon as the camera unit was centered on the corneal apex. All measurements of both eyes were taken successively three times on each device by the same examiner (J.S.) with the room light switched off. The participants were asked to move their heads away from the chin rest between the measurements. The measurements were considered acceptable if they satisfied the quality criteria for each individual device defined by the manufacturer. For the Cirrus HD-OCT, the measurement quality was assessed visually by the examiner. All four devices were equipped with the latest software version available (IOLMaster 700 version 1.5, Pentacam HR version 1.20r87, iDesign version Firmware 1.3 (MX 1.4.1.2), and Cirrus HD-OCT 400 version 7.03.19).

### 2.1. Device Specifications

#### 2.1.1. IOLMaster 700

The IOLMaster 700 is a SS-OCT in combination with a multidot-keratometer. The interferometer is a Mach-Zehnder interferometer [8], and the laser used is a vertical cavity surface-emitting laser (VCSEL) with a very long coherence length. The wavelength can vary from 1035 nm to 1095 nm [8]. The multidot-keratometer comprises 18 points, which are arranged on three rings radially to pivot the instrument. The optical axis of the SS-OCT and multidot-keratometer is identical, to ensure that the B-scan passes through the measuring points. The measurement of CCT occurs at the corneal vertex.

#### 2.1.2. Pentacam HR

The Pentacam HR (OCULUS, Wetzlar) collects data based on the Scheimpflug principle [4]. The light source is a blue light-emitting diode (LED) with a wavelength of 475 nm [9]. The images of the cornea are captured by a 1.45-megapixel camera that records 138000 data points within 2 seconds. Keratometry is calculated using a reference surface. To determine the total refractive power of the cornea, the Pentacam HR uses the formula for thick lenses. For this purpose, a refractive index of the cornea of 1.376 and a refractive index of the aqueous humor in the anterior chamber of 1.336 are used [10].

#### 2.1.3. Cirrus HD-OCT

The Cirrus HD-OCT (Carl Zeiss Meditec AG) is based on the spectral domain OCT (SD-OCT) technology. It takes up to 27000 A-scans per second and has an axial resolution of 5 microns. It can be used both for analysis of retinal structures and for evaluation of the anterior segment. For this purpose, the focus of the beam of the light source is directed onto the cornea. In order to assess the cornea, two modes are available: the anterior segment 5-line raster mode and the anterior segment cube 512 × 128 mode. The 5-line raster mode generates 5 lines on the cornea, with a spacing of 250 microns. Each line is formed by 4096 A-scans. The cube 512 × 128 generated 1024 A-scans in a square on the cornea [11]. In this study, the 5-line raster mode was used.

#### 2.1.4. iDesign Aberrometer

The iDesign advanced wavescan studio (Abbott Medical Optics Inc.) is a Hartmann test-based aberrometer. In a pupil size of 7 mm, 1250 points are captured [12]. The aberrometry results are calculated via a Hartmann-Shack sensor, which bases its measurements on Fourier algorithms. In addition to the aberrometry, the device performs a corneal topography, pupillometry, and iris registration. The advantage of a Hartmann test-based topography is that there are no projected rings on the cornea, which could make establishing between the measuring points difficult, when gauging irregular corneas [13]. Instead of rings, points in shape of a Hartmann pattern are projected onto the corneal surface.

### 2.2. Statistical Analysis

Statistical analysis was performed using IBM-SPSS for Windows software version 23 (International Business Machines Corp., USA). To avoid any bias, we selected randomly only one eye of each subjects as the study eye. To compare the corneal curvature in the Cartesian coordinate system, the keratometry values (flat K, steep K, and steep axis) were converted into Jackson's cross cylinder power vector components (J0 and J45) as described by Thibos et al. [14]. The variables were applied to the following statistical analyses after confirming normality of the data using a one-sample Kolmogorov-Smirnov test: To evaluate the reproducibility of the measurements, the intraclass correlation coefficient (ICC) and its 95% confidence interval (CI) value were calculated. In general, an ICC greater than 0.8 is considered good repeatability of measurements and greater than 0.9 is considered excellent repeatability of measurements. To determine the agreement between the devices, Bland-Altman plot analysis was performed [15]. The 95% limits of agreement (LoA) were estimated by mean difference ± 1.96 × standard deviation (SD) of the differences which provides an interval within which 95% of the differences between measurements are expected to lie [15]. To review the agreement, one-sample *t*-test was performed by setting the test value equal to zero. To detect proportional bias, we used linear regression analysis. A *p* value less than 0.05 was considered statistically significant.

## 3. Results

Fifty-five eyes of 55 subjects were analyzed in this study. The mean age of the subjects was 39.8 ± 13.14 SD (range 19 to 64 years). Thirty-one participants (56%) were female. The mean objective spherical equivalent was −2.21 ± 3.65 SD diopters (D), and the mean cylinder was −0.78 ± 0.62 D. CCT was measured with the IOLMaster 700, Pentacam HR, and Cirrus HD-OCT. The iDesign aberrometer is not capable of measuring CCTs. Keratometry measurements were obtained by the IOLMaster 700, Pentacam HR, and iDesign aberrometer. The Cirrus HD-OCT is not equipped with measuring keratometry values. Table 1 demonstrates all parameters obtained by the four devices.

### 3.1. Central Corneal Thickness (CCT)

#### 3.1.1. Repeatability

The intraoperator repeatability of CCT values of the IOLMaster 700, Pentacam HR, and Cirrus HD-OCT was high (Table 2). The ICCs ranged from 0.995 to 0.999. The mean differences between the first two measurements of each device (IOLMaster 700 (0.09 *μ*m), Pentacam HR (1.65 *μ*m), and Cirrus HD-OCT (0.65 *μ*m)) were low. The confidence intervals of the ICCs and the LoA for each device were narrow. The IOLMaster 700 had the highest ICC and lowest mean difference of the repeated CCT measurements.

#### 3.1.2. Agreement

**Figure 1:**
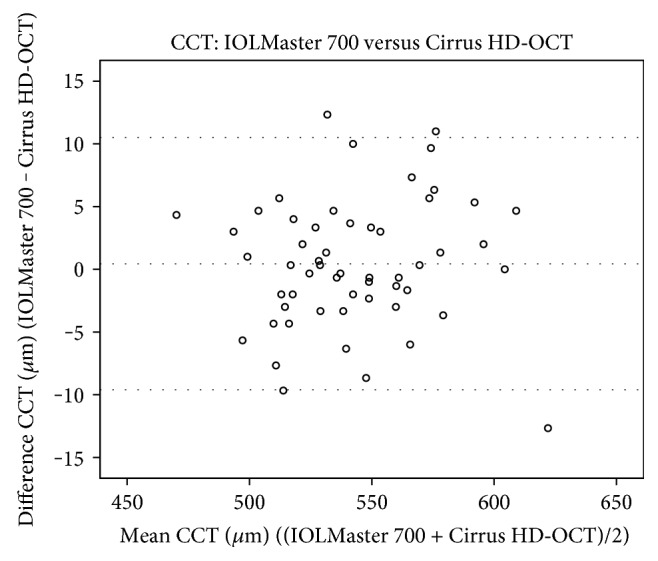
A Bland-Altman plot showing the agreement of CCT measurements between the IOLMaster 700 and Cirrus HD-OCT devices. The line shows the mean difference, and the top and bottom dashed lines show the upper and lower 95% LoA, respectively.

The Pentacam HR exhibited higher OCT values than the IOLMaster 700 (on average 10.99 *μ*m) and Cirrus HD-OCT (on average 11.44 *μ*m) (Table 3). However, the mean difference of CCT values between the IOLMaster 700 and Cirrus HD-OCT was small (0.44 *μ*m). CCT measurements between the Pentacam HR and IOLMaster 700 (*p* < 0.0001) and Pentacam HR and Cirrus HD-OCT (*p* < 0.0001) were statistically significantly different whereas between the IOLMaster 700 and Cirrus HD-OCT, the CCT values were comparable (*p* = 0.519).  Figure 1 shows the Bland-Altman plot for CCT measurements between the IOLMaster and Cirrus HD-OCT with a mean difference of 0.44 *μ*m (95% LoA, 10.5 to −9.6). There was no proportional bias (*p* = 0.619).

### 3.2. Keratometry

#### 3.2.1. Repeatability

The intraoperator repeatability of J0, J45, and Kmean (IOLMaster 700, Pentacam HR, and iDesign) was high (Table 2). The ICCs ranged from 0.974 to 0.999. The mean differences of J0, J45, and Kmean between the first two measurements of each device (IOLMaster 700 (0.02, 0.009, and −0.002, respectively), Pentacam HR (−0.02 for all parameters), and iDesign (−0.007, −0.01, and 0.05, respectively)) were low. The confidence intervals of the ICCs and the LoA for each device were narrow, and the repeated measurement values of J0, J45, and Kmean were comparable (*p* = 0.211 to *p* = 0.066).

#### 3.2.2. Agreement

**Figure 2:**
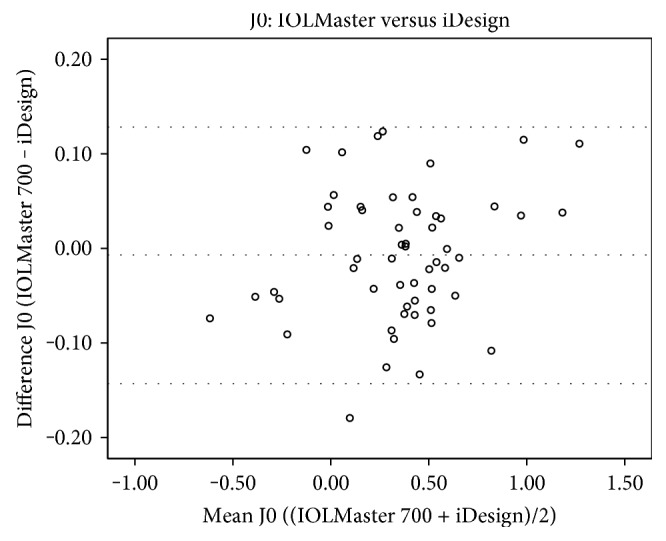
A Bland-Altman plot showing the agreement of J0 vector components between the IOLMaster 700 and iDesign devices. The line shows the mean difference, and the top and bottom dashed lines show the upper and lower 95% LoA, respectively.

**Figure 3:**
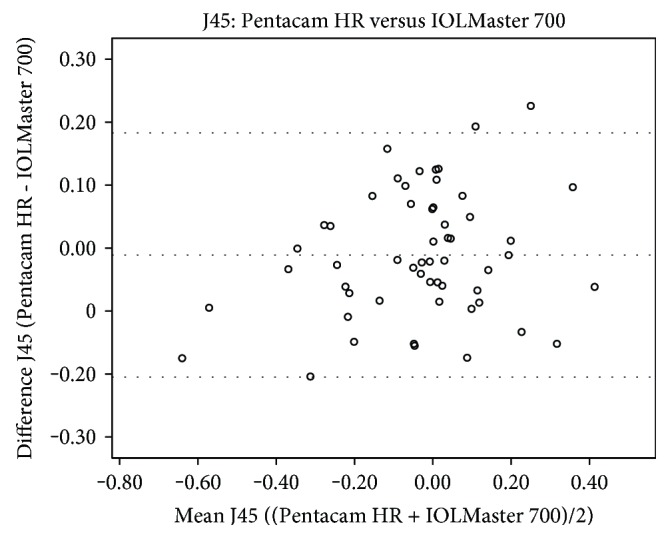
A Bland-Altman plot showing the agreement of J45 vector components between the Pentacam HR and IOLMaster 700 devices. The line shows the mean difference, and the top and bottom dashed lines show the upper and lower 95% LoA, respectively.

**Figure 4:**
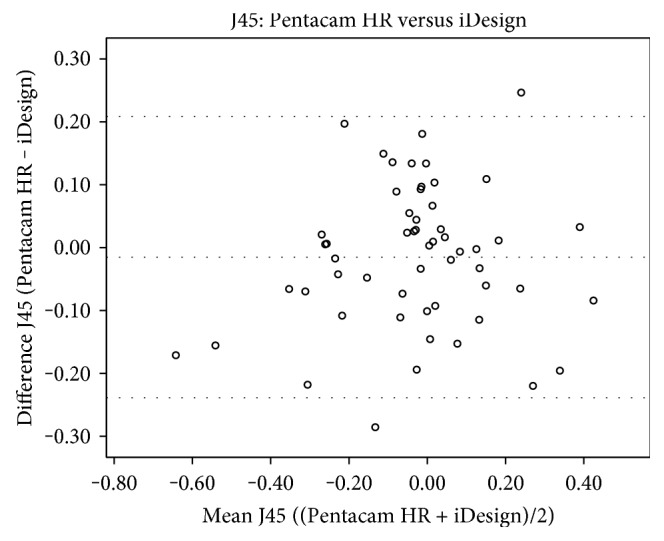
A Bland-Altman plot showing the agreement of J45 vector components between the Pentacam HR and iDesign devices. The line shows the mean difference, and the top and bottom dashed lines show the upper and lower 95% LoA, respectively.

**Figure 5:**
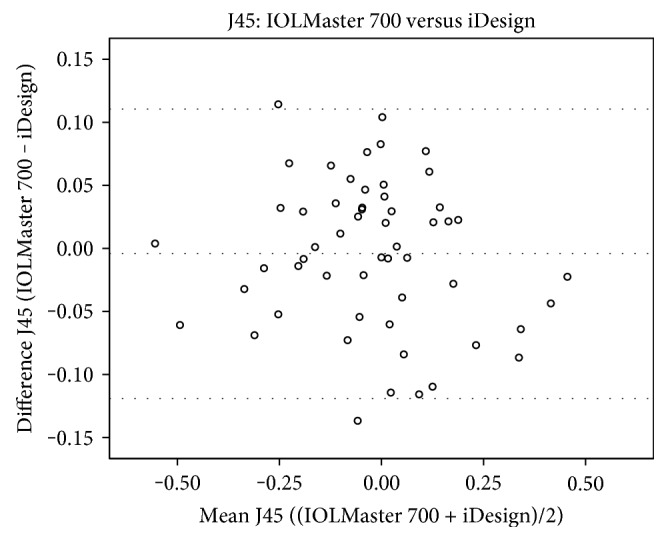
A Bland-Altman plot showing the agreement of J45 vector components between the IOLMaster 700 and iDesign devices. The line shows the mean difference, and the top and bottom dashed lines show the upper and lower 95% LoA, respectively.

J0 readings of the Pentacam HR were statistically significantly higher than those of the IOLMaster 700 (*p* = 0.009) and iDesign (0.041) (Table 4). However, no statistically significant differences of J0 values were found in the comparison between the IOLMaster 700 and iDesign (*p* = 0.426). In addition, there were also no statistically significant differences in any comparison of J45 between the devices Pentacam HR versus IOLMaster 700 (*p* = 0.412), Pentacam HR versus iDesign (*p* = 0.325), and IOLMaster versus iDesign (*p* = 0.591). Kmean, which is an arithmetic mean of Ksteep and Kflat and therefore not in the Cartesian coordinate system, showed statistical significance in all comparisons (*p* < 0.0001 for all device-paired comparisons). Figures 2–5 show the Bland-Altman plots for parameter differences with narrow 95% LoA. The mean difference for J0 between the IOLMaster and iDesign was −0.007 D (95% LoA, 0.1282 and −0.1413), for J45 between the Pentacam HR and IOLMaster 700 was −0.011 D (95% LoA, 0.1829 and −0.2049), for J45 between the Pentacam HR and iDesign was −0.0153 D (95% LoA, 0.2082 and −0.2388), and for J45 between the IOLMaster 700 and iDesign was −0.0042 D (95% LoA, 0.1105 and −0.1191). There was no proportional bias for all these parameter differences (J0: IOLMaster 700 versus iDesign, *p* = 0.125, and J45: Pentacam HR versus IOLMaster, *p* = 0.09; Pentacam HR versus iDesign, *p* = 0.387; and IOLMaster 700 versus iDesign, *p* = 0.284).

## 4. Discussion

Repeatability and consistency of devices are important for both clinical practice and research settings. To our knowledge, this is the first study to comprehensively evaluate the repeatability and comparability of CCT and keratometry measurements with the four devices included in our analysis: Pentacam HR, IOLMaster 700, Cirrus HD-OCT, and iDesign. In summary, the results of our study indicated that the intraoperator repeatability of CCT and keratometry measurements was high for all parameters and all devices analyzed. However, the agreement of CCT values between the Pentacam HR versus IOLMaster 700 and the Pentacam HR versus Cirrus HD-OCT was lacking. The thickness of the central cornea measured by the Pentacam HR was statistically significantly thicker than that measured by the IOLMaster 700 (10.99 *μ*m) and Cirrus HD-OCT (11.44 *μ*m). In addition, there was no agreement of J0 readings between the Pentacam HR versus IOLMaster 700 (*p* = 0.009) and the Pentacam HR versus iDesign (*p* = 0.041). The Kmean showed also no agreement between the devices tested (*p* < 0.0001 of all comparisons). For clinical practice, we therefore conclude that the measurements with no statistical agreement should not be used interchangeably.

CCT values are essential for the preoperative assessment of keratorefractive surgery. The calculation of corrected intraocular pressure also depends on CCT measurements. Our results are in line with the findings of other studies in measuring CCT. Kanellopoulos and Asimellis [16] investigated the agreement between a Scheimpflug imaging system and a spectral domain OCT and found a significant difference of 12.2 ± 10.01 *μ*m between the instruments (*p* = 0.0002). Chen et al. [17] found a significant difference of 10.9 ± 5.93 *μ*m (95% LoA, −0.7 to 22.5 *μ*m) in comparing the Pentacam HR with a Fourier-domain OCT. However, Huang et al. [9] reported a good agreement between the Pentacam HR and the LenStar/Biograph biometer with a mean difference of 3.72 ± 6.10 *μ*m (95% LoA, −8.2 to 15.7 *μ*m) and suggested interchangeable use of these two devices for most practical purposes. Yu et al. [18] studied the agreement between Scheimpflug analyzer (Corvis ST), Pentacam, and an ultrasonic pachymeter and found significantly thicker CCT readings for Pentacam than that for Corvis ST. The mean difference was 3.2 *μ*m (95% LoA, −15.8 to 9.5 *μ*m), and they therefore proposed that the devices can be used interchangeably.

Accurate measurement of corneal astigmatism is mandatory for patients undergoing refractive corneal or lens surgery. To statistically compare the astigmatism of the cornea, keratometery readings were transformed into vector components of J0 and J45. Our results demonstrate statistically significant difference of J0 vector component between the Pentacam HR and IOLMaster 700 as well as the Pentacam HR and iDesign. The Pentacam HR measured in comparison to IOLMaster 700 (0.033 D), and in comparison to iDesign (0.0026 D), higher J0 vector components. According to the study by Read et al. [19], the Pentacam HR also showed higher values for J0 than the Placido topographer. Tajbakhsh et al. [3] demonstrated that the Pentacam HR produces the lowest values for the J0 and J45 vectors compared to the two Placido topographers. The study by Read et al. [19] showed smaller differences for J0, J45, and Kmean. The mean difference of Kmean in their study was 0.12 ± 0.14 D with 95% LoA between 0.4 D and −0.17 D. Accordingly, the Pentacam HR and the Medmont E300 for keratometry were reported as interchangeable in their study. The different results arise from the general comparison of different optical methods. Prakash et al. [20] compared a Placido-based method with the Pentacam HR, while in our study, the Pentacam HR was compared to a multidot-keratometer. The study by Tajbakhsh et al. [3] compared the Pentacam HR with two Placido-based methods, and the Pentacam HR was regarded as interchangeable with the TMS-4 topographer, as the differences in keratometry were not considered clinically relevant. Prakash et al. [20] compared the iDesign with a Scheimpflug-Placido combination and suggested that these two devices should not be used interchangeably. The mean differences were below 0.1 D and, thus, lower than that in our study. However, the LoA between Scheimpflug-Placido method and the iDesign were greater than 1 D. The values that are used for the calculation of IOL are very sensitive, which is why the devices should not be used interchangeably [20]. Dong et al. [21] reported that the Pentacam HR and IOLMaster 500 were not interchangeable. This study was carried out in two groups. There were normal eyes and highly myopic eyes, with a spherical equivalent over −6 D. In the group of normal eyes, the differences for the steep keratometry and Kmean were statistically significant (*p* < 0.05). In the group of highly myopic eyes, only the Kmean for the Pentacam HR and IOLMaster 500 was significantly different. All other keratometry data had no significant difference. In both groups, the keratometry values of the Pentacam HR were lower than that of the IOLMaster 500. The 95% LoA for the group of normal eyes and for the group of highly myopic eyes were larger than 1 D. They proposed that the devices should not be used interchangeably. The results of our study are comparable to those of Dong et al. for the group of normal eyes. However, the IOLMaster 500 is a multidot-keratometer with 6 points and the IOLMaster 700 measures the corneal curvature with 18 points. Due to the greater number of points, it is likely that the IOLMaster 700 measures values that are closer to the “true value” of corneal curvature.

The reason for statistically significant differences could be the different measuring methods. The cornea is not spherical [17]. Differences result from variable measuring points. The measurement of the Cirrus HD-OCT is performed manually using the caliper tool and is, thus, dependent on the skill of the examiner. The measuring point must be optically selected and is, therefore, not precisely defined. For these reasons, the high repeatability of the Cirrus HD-OCT is surprising. The difference between the Pentacam HR and IOLMaster 700 could result from the different algorithms that are used for corneal thickness calculation. Another important factor is the fixation. The quicker a measurement can be taken, the less it depends on the fixation of the subject. The impact of the tear film on the measurements should also be taken into account.

Differences in keratometry readings of the three devices could be justified by the different densities of the measuring points. The Pentacam HR captures 138000 data points; the iDesign, 1250; and the IOLMaster 700, 18.

All devices demonstrated excellent repeatability in measuring CCT and keratometry readings. Similar results were reported by Wang et al. [22]. Read et al. [19] examined the repeatability and reproducibility of the Pentacam HR in comparison to Medmont E300 (Placido-based method). The ICC of J0 and J45 for Pentacam HR was greater than 0.9, which corresponds to the results of our work.

Potential limitations of our study are, firstly, the relatively small sample size (55 eyes) and, secondly, our population consisting of only healthy volunteers with normal corneas. Therefore, in further studies, these devices should be examined in patients with cataract and keratoconus or after undergoing any corneal refractive surgery.

In conclusion, our data suggest that the clinician should be aware of significant differences of CCT and keratometry values when measuring with different devices. In clinical settings where CCT values are crucial, we suggest that the CCT results of the Pentacam HR versus IOLMaster 700 and the Pentacam HR versus Cirrus HD-OCT should not be used interchangeably. However, our data suggest that the IOLMaster 700 and Cirrus HD-OCT have good concordance and can be used interchangeably to measure CCT values. However, our results demonstrate that J0 of the Pentacam HR versus IOLMaster 700 and the Pentacam versus iDesign as well as Kmean of the Pentacam HR, IOLMaster 700, and iDesign should also not be used interchangeably.

## Figures and Tables

**Table 1:** CCT and keratometry measurements from all 4 devices.

Parameter	Device			
	IOLMaster 700	Pentacam HR	iDesign	Cirrus HD-OCT
CCT [*μ*m]				
Mean ± SD	543.20 ± 31.69	554.19 ± 26.86	—	542.75 ± 31.34
Range	472–616	484–614		468–628
J0 [D]				—
Mean ± SD	0.350 ± 0.367	0.384 ± 0.342	0.358 ± 0.353	
Range	−0.65–1.32	−0.51–1.18	−0.58–1.21	
J45 [D]				—
Mean ± SD	−0.027 ± 0.197	−0.385 ± 0.218	−0.023 ± 0.205	
Range	−0.55–0.44	−0.73–0.41	−0.56–0.47	
Kmean [D]				—
Mean ± SD	42.76 ± 1.49	43.25 ± 1.51	43.52 ± 1.54	
Range	38.89–45.90	39.42–46.43	39.54–46.65	

CCT: central corneal thickness; J0: astigmatism 90/180 degrees; J45: astigmatism 45/135 degrees; Kmean: mean keratometry; —: no data available because of device specifications.

**Table 2:** Intraoperator repeatability measurements.

Parameter/device	ICC	95% confidence interval	Difference^a^ (mean ± SD)	95% LoA	*p* value
CCT [*μ*m]					
IOLMaster 700	0.999	0.998–0.999	−0.09 ± 2.76	−0.83–0.65	0.808
Pentacam HR	0.995	0.992–0.997	−0.25 ± 5.18	−1.65–1.14	0.717
Cirrus HD-OCT	0.995	0.992–0.997	0.91 ± 5.79	−0.65–2.47	0.250
J0 [D]					
IOLMaster 700	0.989	0.983–0.993	0.02 ± 0.11	−0.01–0.04	0.213
Pentacam HR	0.986	0.978–0.991	−0.02 ± −0.12	−0.05–0.01	0.252
iDesign	0.994	0.991–0.996	−0.007 ± 0.07	−0.007–0.01	0.468
J45 [D]					
IOLMaster 700	0.974	0.959–0.984	0.009 ± 0.08	−0.01–0.03	0.406
Pentacam HR	0.984	0.975–0.990	−0.02 ± 0.06	−0.03–0.001	0.066
iDesign	0.977	0.963–0.986	−0.01 ± 0.08	−0.03–0.01	0.304
Kmean [D]					
IOLMaster 700	0.999	0.999–1.0	−0.002 ± 0.09	−0.03–0.02	0.842
Pentacam HR	0.999	0.999–1.0	−0.02 ± 0.08	−0.03–0.005	0.145
iDesign	0.992	0.987–0.995	0.05 ± 0.29	−0.03–0.13	0.211

CCT: central corneal thickness; J0: astigmatism 90/180 degrees; J45: astigmatism 45/135 degrees; Kmean: mean keratometry; ICC: intraclass correlation coefficient; LoA: limits of agreement; ^a^between the second measurement of each eye; *p* values were calculated by one-sample *t*-test.

**Table 3:** CCT measurement differences between the three devices.

Parameter	Device comparison		
	Pentacam HR versus IOLMaster 700	Pentacam HR versus Cirrus HD-OCT	IOLMaster 700 versus Cirrus HD-OCT
CCT [*μ*m]			
Difference^a^ (mean ± SD)	10.99 ± 7.57	11.44 ± 8.91	0.44 ± 5.12
Range	−11.33–24.67	−24.00–26.67	−12.67–12.33
95% CI	8.94–13.04	9.03–13.85	−0.93–1.83
*p* value	<0.0001	<0.0001	0.519

CCT: central corneal thickness; CI: confidence interval; ^a^between the mean measurements of each device and of each eye; *p* values were calculated by one-sample *t*-test.

**Table 4:** Keratometry measurement differences between the three devices.

Parameter	Device comparison		
	Pentacam HR versus IOLMaster 700	Pentacam HR versus iDesign	IOLMaster 700 versus iDesign
J0 [D]			
Difference^a^ (mean ± SD)	0.033 ± 0.09	0.026 ± 0.09	−0.007 ± 0.07
Range	−0.25–0.27	−0.16–0.31	−0.18–0.12
95% CI	0.009–0.056	0.01–0.05	−0.03–0.01
*p* value	0.009	0.041	0.426
J45 [D]			
Difference^a^ (mean ± SD)	−0.011 ± 0.10	−0.015 ± 0.11	0.004 ± 0.06
Range	−0.20–0.23	−0.29–0.25	−0.14–0.11
95% CI	−0.01–0.04	−0.01–0.05	−0.01–0.02
*p* value	0.412	0.325	0.591
Kmean [D]			
Difference^a^ (mean ± SD)	0.49 ± 0.11	−0.27 ± 0.26	−0.76 ± 0.25
Range	0.27–0.91	−1.34–0.26	−1.95 to −0.24
95% CI	0.46–0.52	−0.34 to −0.20	−0.83 to −0.69
*p* value	<0.0001	<0.0001	<0.0001

J0: astigmatism 90/180 degrees; J45: astigmatism 45/135 degrees; Kmean: mean keratometry; CI: confidence interval; ^a^between the mean measurements of each device and of each eye; *p* values were calculated by one-sample *t*-test.
